# Suspected poor-quality medicines in Kenya: a retrospective descriptive study of medicine quality-related complaints reports in Kenya’s pharmacovigilance database

**DOI:** 10.1186/s12889-024-20036-4

**Published:** 2024-09-19

**Authors:** Anthony Martin Toroitich, Rachel Armitage, Sangeeta Tanna

**Affiliations:** 1 Pharmacy and Poisons Board, P.O. Box 27663 – 00506, Nairobi, Kenya; 2 Leicester School of Pharmacy, De Montfort University, The Gateway, Leicester, LE1 9BH UK; 3 School of Archaeology and Ancient History, University of Leicester, University Road, Leicester, LE1 7RH UK

**Keywords:** Poor-quality medicine, Falsified medicine, Substandard medicine, Post-market surveillance, Therapeutic failure, Adverse drug reaction, Patient safety, Africa

## Abstract

Poor-quality, substandard and falsified, medicines pose a significant public health threat, particularly in low-middle-income countries. A retrospective study was performed on Kenya's Pharmacovigilance Electronic Reporting System (2014–2021) to characterize medicine quality-related complaints and identify associations using disproportionality analysis. A total of 2767 individual case safety reports were identified, categorized into medicines with quality defects (52.1%), suspected therapeutic failure (41.6%), and suspected adverse drug reactions (6.3%). Predominantly reported were antineoplastic agents (28.6%), antivirals (11.7%), and antibacterial agents (10.8%) potentially linked to non-adherence to good manufacturing practices, inappropriate usage and supply chain degradation. Notably, analgesics (8.2%), and medical devices (3.5%) notified had quality defects, predominantly from government health facilities (60.0%). Antineoplastic agents (20.2%) and antivirals (3.7%) were frequently reported from suspected therapeutic failures and suspected adverse drug reactions, respectively, across both private for-profit facilities (26.5%) and not-for-profit facilities (5.4%). Underreporting occurred in unlicensed health facilities (8.1%), due to unawareness and reporting challenges. Pharmacists (46.1%), and pharmaceutical technicians (11.7%) predominantly reported quality defects, while medical doctors (28.0%) reported suspected therapeutic failures. Orally administered generic medicines (76.9%) were commonly reported, with tablets (5.8%) identified as potential sources of suspected adverse drug reactions, while quality defects were notified from oral solutions, suspensions, and syrups (7.0%) and medical devices (3.9%). The COVID-19 pandemic correlated with reduced reporting possibly due to prioritization of health surveillance. This study provides valuable evidence to supporting the use of medicine quality-related complaints for proactive, targeted regulatory control of high-risk medicines on the market. This approach can be strengthened by employing standardized terminology to prioritize monitoring of commonly reported suspected poor-quality medicines for risk-based sampling and testing within the supply chain.

## Background

Effective healthcare service delivery hinges upon the presence of robust healthcare systems that ensure accessible, affordable, and availability of high-quality medicines for all. In this study, an expanded definition of the term 'medicine' is adopted to encompass a broad spectrum of medicinal products, including medicines, health products, medical devices, and health technologies [1]. The World Health Organization (WHO) estimates that globally 10% of medical products are substandard and falsified (SF) medicines [2], however, reports indicate a higher prevalence of such poor-quality medicines in some regions [3]. Substandard medicines refer to authorised products that fail to meet the quality standards or specifications [4] as set by either regulatory authorities or the manufacturers. Substandard medicines may contain incorrect or insufficient quantities of active pharmaceutical ingredients (APIs), toxic impurities/contaminants, they may be degraded, or may be manufactured under inadequate subpar quality assurance conditions. Degraded medicines are products that become substandard after manufacturing resulting from storage, mishandling, or transportation within their designated shelf life [5]. Falsified medicines are intentionally misrepresented with regard to their identity, composition, or source and may contain no or the incorrect API, or toxic ingredients or the wrong amount of the correct API. They are often fraudulently produced and labelled to closely mimick genuine products [4]. Both generic and branded innovator medicines have emerged as prime targets for being substandard or falsified [4]. SF medicines presents challenges to regulators, and poses severe risks to patients’ health and compromise the resilience of healthcare systems.

A meta-analysis by Ozawa et. al., 2018 observed a prevalence of about 13.6% of essential medicines sampled and tested in low medium income countries (LMICs) as being substandard or falsified [6]. No studies have been conducted to determine the prevalence of SF in Kenya. However, Thoithi and colleagues reported an overall prevalence of substandard medicines ranging from 6.1 to 21.1% from 2001 – 2010 [7, 8]. A study conducted in Kenyan capital city, Nairobi, found out that about 37.7% of sampled Amoxicillin formulations failed to comply the pharmacopeial specifications [9]. The estimates however are based on poorly designed medicine quality surveys, most of which have numerous limitations, such as insufficient or inadequate samples, inconsistent sampling methods, and variable types of analytical testing methods [10]. The WHO global surveillance and monitoring system for SF medicines still estimates this is a small portion of the entire problem with most cases remaining unreported [11]. SF medicines are rarely efficacious and often lead to disastrous health consequences including treatment failure [12], disability and death [6] and hinder disease management by compromising patient outcomes. Additionally, they exacerbate drug resistance [2], lead to serious adverse drug reaction [12], engender unintentional medication non-adherence [3], erode public trust in healthcare systems, cause wastage of valuable scarce resources, and compound the economic burden of a nation [6].

The regulation of medicines in Kenya is under the mandate of the Pharmacy and Poisons Board (PPB) [13], which is responsible for ensuring the elimination of poor-quality medicines in the nation’s pharmaceutical supply chain. To facilitate this onus duty, among other regulatory functions, the PPB has implemented an innovative online pharmacovigilance tool, the Pharmacovigilance Electronic Reporting System (PvERS), which serves as an integral component of its national post-market surveillance system. The PvERS is utilized for the notification of individual case safety reports (ICSRs) pertaining to complaints arising from inadvertent exposure to suspected poor-quality medicines and suspected adverse drug events (sADEs). Thus, PvERS fosters passive surveillance by healthcare professionals, patients, and members of the public, enabling the direct identification and reporting of suspected poor-quality medicines within the pharmaceutical supply chain in Kenya. The term “ICSRs” refers to a valuable data source originating from an individual record in the PvERS as an sADEs, suspected poor-quality medical products (sPQMs), adverse events following immunization (AEFIs), medication errors, adverse incidences usage from medical devices, and suspected blood products reactions [14]. The term “sADEs” is used to describe any unfavourable medical incident observed in an individual exposed to a specific medicine, irrespective of appropriateness, which may or may not have a causal relationship with the medicine [15]. sADEs is a broader definition in comparison to suspected adverse drug reactions (sADRs) which are harmful and unintended responses to a medicine when used appropriately at a normal dosage [16]. The term, sPQMs, describes the non-compliance of a medicine with quality affecting organoleptic appearance or microbiological properties, as well as labelling or packaging information. The term, AEFIs, refer to untoward incidents that occur after a vaccination has been administered, without a causal relationship to the vaccines. These events can manifest as abnormal laboratory findings, unfavorable or unintended signs, symptoms, or diseases [17]. A medication error refers to any preventable incident that might result in the improper use of a medicine or patient injury while under the management of a healthcare professional or patient [18].

Despite the wealth of data within the PvERS database, there has not been an in-depth analysis of ICSRs in the context of suspected poor-quality medicines. This study represents the first comprehensive analysis of ICSRs in the context of medicine quality-related complaints (MQRCs) in Kenya. The study aims to identify and analyse distinct patterns and trends in MQRCs to facilite the early detection and targeted post-market surveillance of suspected poor-quality medicines.

## Methods

### Study design

This retrospective descriptive study aimed to analyse all ICSRs that relate to suspected poor-quality medicines in Kenya’s PvERS from January 2014 to December 2021. Data collection and extraction period occurred between November 2021 and January 2022. This data focused on the types, frequency, and sources of documented complaints to determine the categories and incidences of MQRCs.

### Study setting and data source

The study utilized Kenya’s national ICSR database, PvERS which contained over 16,000 ICSRs as of June 2022. The database captures information such as health facility, reporter details, county location of the ICSR, reported quality issues, and product-specific data like brand and generic names, batch number, manufacture and expiry dates, storage conditions, and manufacturer and supplier information. Additionally, the sADRs dataset also includes anonymized patient information and specifics about the reaction.

### Identification and characterization of MQRCs from ICSRs

**Fig. 1:**
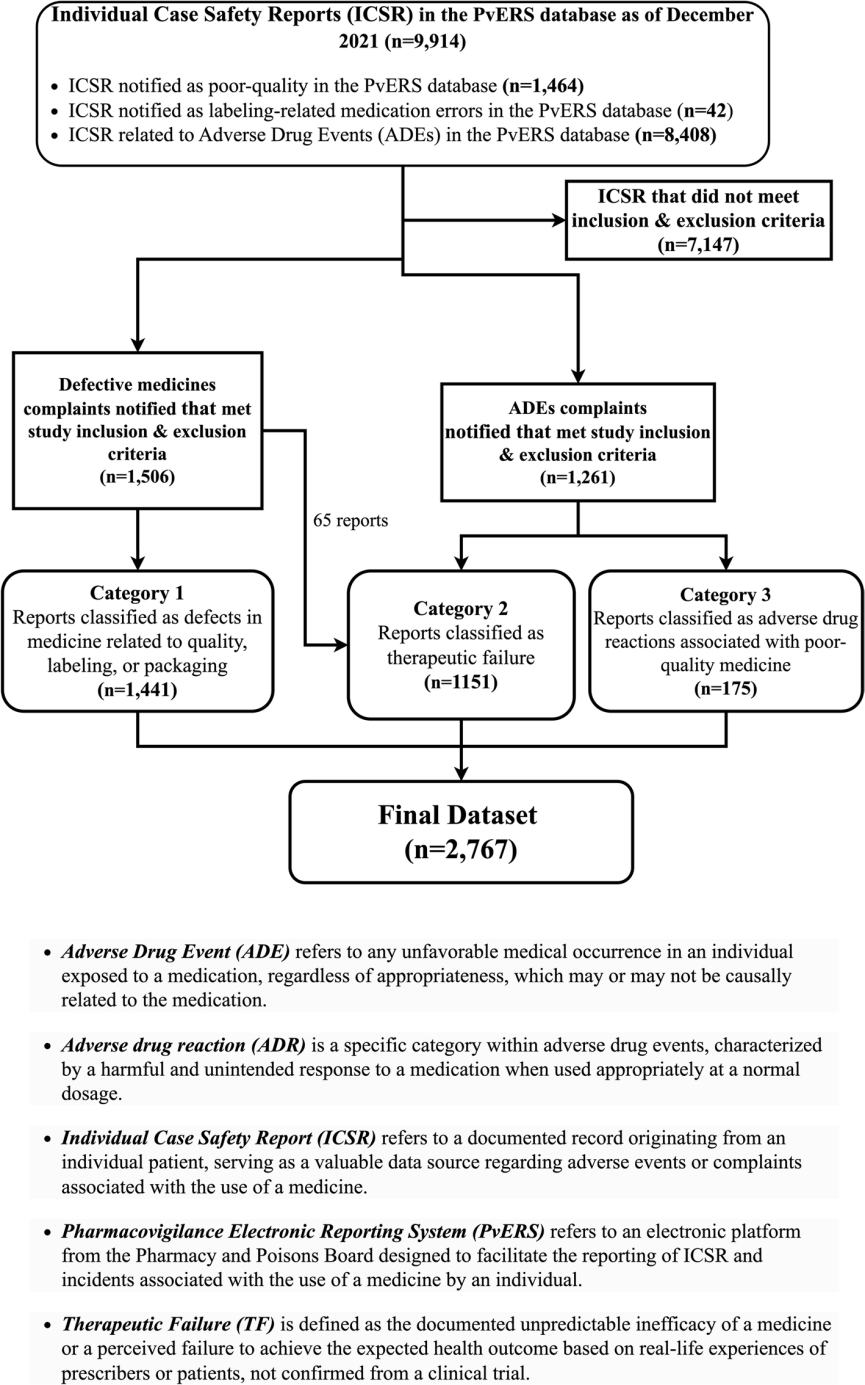
Flowchart depicting data extraction of medicine-quality related complaints from Kenya’s PvERS pharmacovigilance database

For this study, 9,914 ICSRs encompassing all reports received between January 2014 and December 2021 were considered. The ICSRs recorded as sADRs, sPQMs, medication errors, and adverse incidences usage from medical devices were considered for this study because they significantly contribute to MQRCs as shown in Fig. 1 below. The data extracted was recorded in a Microsoft Excel spreadsheet. Prior to analysis, each MQRCs was pre-processed and redacted to remove personal or manufacturer identifiers, eliminate duplicate entries, address any instances of missing or incomplete data, and resolve data inconsistencies. To ensure the integrity and reliability of the dataset, duplicate reports with replicate information based on the name of the medicine, ICSRs, county source of origin of the report, date reported, and patient details were manually eliminated. This approach was chosen to maintain a consistent and reliable dataset, minimizing the risk of data inconsistencies and potential errors during the analysis.

The dataset was subjected to a specified inclusion and exclusion criteria before categorisation into three distinct groups as summarized in Table 1. These three categories were: suspected medicines with quality defects (sMQDs) (category 1); suspected poor-quality medicines that resulted in therapeutic failure (sTF) related to their experience of using a medicine (category 2); and sADRs reports suspected to be due to poor-quality medicines (category 3). sTF is defined as a documented unpredictable inefficacy of a medicine or a perceived failure to achieve the expected health outcome based on real-life experiences of prescribers or patients not confirmed from a clinical trial [19].

**Table 1:** Criteria for identifying and categorizing MQRCs in individual case safety reports

Complaints category	Inclusion criteria	Exclusion criteria
General inclusion criterion	All ICSR entries in PvERS database that had included either a trade name of the medicine or the APIs of the medicine.	Incomplete data ICSR entries that neither had a product trade name nor APIs name stated.
		Reports identified as duplicate entries.
	All ICSR entries in PvERS database captured between 1^st^ January 2014 – 31^st^ December 2021	All ICSR entries indicated with the word, ‘Test’ captured during PvERS user system development and validation
Category 1: sMQDs	Packaging-related problems (e.g., labelling errors, lot or batch identifier issues, illegible label information, leakages)	All medicine quality related complaint entries not in categories 2 & 3
	Manufacturing-related problems (e.g., Incomplete pack, packaging quantity issue)	
	Formulation-related problems (e.g., product powdering, caking, phase separation, clumping, sticking, damaged tablets/blisters: broken tablets)	
	Contamination issues (e.g., presence of particles)	
	Environmental degradation (e.g., moulding, colour change, door change, product instability)	
	Medication errors related with product labelling	
	Patients use-related problems or preferences (e.g., abnormal taste and/or odour, opening issues)	
	Other unspecified medicine quality defect, compliant or physical issue	
Category 2: sTF	Reports of suspected poor-quality medicines notified as therapeutic failure	All medicine quality related complaint medicine entries not in categories 1 & 3
Category 3: sADRs	Reports of suspected poor-quality medicines notified as sADRs	All ADRs entries not in categories 1 & 2

### Data analysis

The distribution and characteristics each ICSR meeting the inclusion criteria was analyzed for the type of complaint, route of administration, dosage form, branded or generic medicine status, the complainants’ background (healthcare professional or members of the public), the healthcare facility source that notified the complaint (public or private), the level of the healthcare facility within Kenya’s healthcare system, and the county of origin in Kenya. The analysis focused on several parameters: differentiation between generic and branded innovator medicines; categories of therapeutic classes notified as MQRCs; rates of different categories of MQRCs; sources of MQRCs within various levels of the Kenyan healthcare system; types of dosage forms notified as MQRCs; the influence of the COVID-19 pandemic on the notification of MQRCs; and the geographical origin of the MQRCs in Kenya. The data was presented in tables and appropriate graphics.

### Statistical analysis

A disproportionality analysis was performed using the Reporting Odds Ratio (ROR) which was employed to evaluate the association between the MQRC category and the medicine of interest. The contingency table presented in Table 2 [20] was used to calculate ROR values, with a significant suspected poor-quality medicine signal defined by ROR values ≥2.0 and a *p*-value <0.05 considered statistically significant.

**Table 2:** Contingency table for disproportionality analyses of MQRCs [20]

	**Specified MQRCs category**	**Other MQRCs categories**
**Medicine of interest in PvERS database notified as MQRCs**	a	b
**Other medicines in PvERS database notified as MQRCs**	c	d
**Total medicines in PvERS database notified as MQRCs**	**a + c**	**b + d**

The disproportionality analysis was applied are as follows:

$$\mathbf{R}\mathbf{O}\mathbf{R}= \frac{{\varvec{a}}{\varvec{d}}}{{\varvec{b}}{\varvec{c}}}$$

Where:

a = represents the number of reports of the medicine of interest with specified MQRC category of interest

b = denotes the number of reports of the medicine of interest with other MQRC categories

c = signifies the number of reports of all other medicines with the specified MQRC category of interest in the PvERS database

d = indicates the number of reports of all other medicines with other MQRC categories in the PvERS database

### Ethical considerations

Authority to analyse the data in the PvERS database was granted by the PPB. The ICSR extracted data was redacted and uniquely coded to omit information that may identify a person, brand name of the medicine product, and a health facility.

## Results

### Therapeutic classes and categories of notified as MQRCs

A total of 9,914 ICSRs were documented in the PvERS database between 2014 and 2021. Figure 1 illustrates that the majority (84.8%; *n*=8,408) of ICSRs were classified as ADEs, while a smaller proportion (15.2%; *n*=1,506) were ascribed to MQRCs. Out of 2,767 MQRCs that fulfilled the inclusion and exclusion criteria, medicines with quality defects (category 1) accounted for 52.1%, suspected sPQMs attributed to sTF (category 2) constituted 41.6%, and suspected sPQMs attributed to sADRs (category 3) accounted for 6.3%. No reports of falsified medicines were reported in the database. The results in Table 3 highlight a higher frequency of MQRCs reporting from generic medicines (66.1%; *n*=1829) compared to branded innovator medicines (29.8%; *n*=825). Branded medicines showed a significant notification from sMQDs (ROR:10.2; *p*-value:<0.001), while generic medicines were reported as sADRs (ROR:11.7; *p*-value:<0.001).

**Table 3:** Disproportionality analysis of poor-quality signals by notified medicine version and medicine quality-related complaint category

Version of medicines	Disproportionality measure	Medicine notified as suspected medicine quality defect (sMQD) complaint	Medicine notified as suspected therapeutic failure (sTF) complaint	Medicine notified as suspected adverse drug reactions (sADRs) complaint
**Branded**	No of reports	325	494	6
	Frequency	0.1	0.2	0.0
	ROR (95% CI)	10.2 (8.1-12.9)	2.9 (2.47-3.45)	0.1 (0.0-0.2)
	*p*-value (non-exact)	<0.001	<0.001	<0.001
**Generic**	No of reports	1015	647	167
	Frequency	0.4	0.2	0.1
	ROR (95% CI)	1.5 (1.3-1.8)	0.5 (0.4-0.6)	11.7 (5.7-23.9)
	*p*-value (non-exact)	<0.001	<0.001	<0.001
**Not applicable or not stated**	No of reports	101	10	2
	Frequency	0.0	0.0	0.0
	ROR (95% CI)	8.3 (4.5-15.1)	0.1 (0.1-0.3)	0.3 (0.1-1.1)
	*p*-value (non-exact)	0.1-0.05	<0.001	0.1

A diverse array of therapeutic classes, spanning both communicable and non-communicable diseases (NCDs), were reported as MQRCs. The study identified the top seven classes notified MQRCs to be antineoplastics primarily reported from imatinib; antivirals predominantly reported from tenofovir containing products; antibacterial agents mainly reported from amoxicillin containing products; analgesics; antihypertensives; medical devices; and antiprotozoals, as summarized in Table 4.

**Table 4:** Common therapeutic classes notified as MQRCs, their incidence, and active pharmaceutical ingredients

**Therapeutic class**	**sMQDs**		**sTF**		**sADRs**		**Total**	
**Antineoplastic agents**	*n*=227	8.2%	*n*=558	20.2%	*n*=5	0.2%	*n*=790	28.6%
Imatinib	*n*=213	7.7%	*n*=551	19.9%	*n*=4	0.1%	*n*=768	27.8%
Fluorouracil	*n*=6	0.2%	*n*=0	0.0%	*n*=0	0.0%	*n*=6	0.2%
Cyclophosphamide	*n*=2	0.1%	*n*=1	0.0%	*n*=0	0.0%	*n*=3	0.1%
Ifosfamide + Mesna	*n*=0	0.0%	*n*=2	0.1%	*n*=0	0.0%	*n*=2	0.1%
Other Antineoplastic agents	*n*=6	0.2%	*n*=4	0.1%	*n*=1	0.0%	*n*=11	0.4%
**Antivirals**	*n*=133	4.8%	*n*=89	3.2%	*n*=101	3.7%	*n*=323	11.7%
Tenofovir containing products	*n*=86	3.1%	*n*=32	1.2%	*n*=64	2.3%	*n*=182	6.6%
Efavirenz	*n*=4	0.1%	*n*=19	0.7%	*n*=14	0.5%	*n*=37	1.3%
Zidovudine containing products	*n*=15	0.5%	*n*=7	0.3%	*n*=5	0.2%	*n*=27	1.0%
Dolutegravir	*n*=0	0.0%	*n*=19	0.7%	*n*=5	0.2%	*n*=24	0.9%
Atazanavir containing products	*n*=12	0.4%	*n*=2	0.1%	*n*=2	0.1%	*n*=16	0.6%
Other antivirals	*n*=16	0.6%	*n*=10	0.4%	*n*=11	0.4%	*n*=37	1.3%
**Antibacterial agents**	*n*=175	6.3%	*n*=111	4.0%	*n*=12	0.4%	*n*=298	10.8%
Amoxicillin containing products	*n*=51	1.8%	*n*=37	1.3%	*n*=1	0.0%	*n*=89	3.2%
Co-trimoxazole	*n*=42	1.5%	*n*=6	0.2%	*n*=5	0.2%	*n*=53	1.9%
Gentamicin	*n*=7	0.3%	*n*=30	1.1%	*n*=3	0.1%	*n*=40	1.5%
Flucloxacillin	*n*=15	0.5%	*n*=8	0.3%	*n*=0	0.0%	*n*=23	0.8%
Ceftriaxone	*n*=14	0.5%	*n*=5	0.2%	*n*=0	0.0%	*n*=19	0.7%
Other antibacterial agents	*n*=46	1.7%	*n*=25	0.9%	*n*=3	0.1%	*n*=74	2.7%
**Analgesics**	*n*=203	7.3%	*n*=24	0.9%	*n*=1	0.0%	*n*=228	8.2%
Paracetamol containing products	*n*=169	6.1%	*n*=9	0.3%	*n*=0	0.0%	*n*=178	6.4%
Ibuprofen	*n*=8	0.3%	*n*=11	0.4%	*n*=0	0.0%	*n*=19	0.7%
Diclofenac	*n*=16	0.6%	*n*=0	0.0%	*n*=0	0.0%	*n*=16	0.6%
Tramadol	*n*=6	0.2%	*n*=0	0.0%	*n*=0	0.0%	*n*=6	0.2%
Aspirin containing products	*n*=4	0.1%	*n*=0	0.0%	*n*=1	0.0%	*n*=5	0.2%
Other analgesics	*n*=0	0.0%	*n*=4	0.1%	*n*=0	0.0%	*n*=4	0.1%
**Antihypertensives**	*n*=86	3.1%	*n*=42	1.5%	*n*=18	0.7%	*n*=146	5.3%
Losartan containing products	*n*=22	0.8%	*n*=8	0.3%	*n*=7	0.3%	*n*=37	1.3%
Furosemide	*n*=11	0.4%	*n*=11	0.4%	*n*=0	0.0%	*n*=22	0.8%
Amlodipine containing products	*n*=13	0.5%	*n*=3	0.1%	*n*=3	0.1%	*n*=19	0.7%
Nifedipine	*n*=9	0.3%	*n*=3	0.1%	*n*=3	0.1%	*n*=15	0.5%
Methyldopa	*n*=5	0.2%	*n*=8	0.3%	*n*=0	0.0%	*n*=13	0.5%
Other antihypertensives	*n*=26	0.9%	*n*=9	0.3%	*n*=5	0.2%	*n*=40	1.5%
**Medical** **devices**	*n*=108	3.9%	*n*=14	0.5%	*n*=3	0.1%	*n*=125	4.5%
**Antiprotozoals**	*n*=60	2.2%	*n*=39	1.4%	*n*=1	0.0%	*n*=100	3.6%
Metronidazole	*n*=40	1.5%	*n*=23	0.8%	*n*=0	0.0%	*n*=63	2.3%
Artemisinin antimalarials	*n*=17	0.6%	*n*=14	0.5%	*n*=1	0.0%	*n*=32	1.2%
Sulfadoxine + Pyrimethamine	*n*=1	0.0%	*n*=1	0.0%	*n*=0	0.0%	*n*=2	0.1%
Aminosidine (Paromomycin)	*n*=1	0.0%	*n*=0	0.0%	*n*=0	0.0%	*n*=1	0.0%
Secnidazole	*n*=0	0.0%	*n*=1	0.0%	*n*=0	0.0%	*n*=1	0.0%
Tinidazole	*n*=1	0.0%	*n*=0	0.0%	*n*=0	0.0%	*n*=1	0.0%
**Gynaecological** **agents**	*n*=61	2.2%	*n*=24	0.9%	*n*=2	0.1%	*n*=87	3.1%
**Antimycobacterial** **agents**	*n*=33	1.2%	*n*=48	1.7%	*n*=6	0.2%	*n*=87	3.1%
**Blood** **and** **perfusion** **solutions**	*n*=29	1.1%	*n*=57	2.1%	*n*=0	0.0%	*n*=86	3.1%
**Anaesthetics**	*n*=53	1.9%	*n*=5	0.2%	*n*=0	0.0%	*n*=58	2.1%
**Mineral** **supplements** **+** **vitamins**	*n*=28	1.0%	*n*=31	1.1%	*n*=4	0.1%	*n*=63	2.3%
**Drugs** **used** **in** **diabetes**	*n*=8	0.3%	*n*=18	0.7%	*n*=1	0.0%	*n*=27	1.0%
**Vaccines**	*n*=16	0.6%	*n*=6	0.2%	*n*=0	0.0%	*n*=22	0.8%
**Other** **therapeutic** **products**	*n*=221	8.0%	*n*=85	3.1%	*n*=21	0.8%	*n*=327	11.8%
**Total**	*n*=1441	52.1%	*n*=1151	41.6%	*n*=175	6.3%	*n*=2767	100.0%

Table 5 illustrates the reporting rates of sMQDs (category 1) in the PvERs database. The predominantly reported for analgesics (7.3%; ROR:8.5; *p*-value < 0.001), medical devices (3.9%; ROR:6.2; *p*-value<0.001), gynaecological agents (2.2%; ROR:2.2; *p*-value<0.001), and anaesthetics (1.9%; ROR:10.1; *p*-value<0.001), highlighting these therapeutic classes as particularly vulnerable.

**Table 5:** Reporting rates of MQRCs in Kenya’s PvERS database attributed to sMQDs

**Therapeutic class**	**ROR (95% CI)**	***p*-value (non-exact)**
**Antineoplastics**	0.3 (0.21-0.30)	<0.001
Imatinib	0.2 (0.20-0.29)	0.001
Fluorouracil	∞ (N/A)	0.2-0.1
Cyclophosphamide	1.3 (0.11-13.89)	0.975-0.2
Ifosfamide + Mesna	0 (N/A)	0.975-0.2
Other antineoplastic agents	0.8 (0.23-2.48)	0.975-0.2
**Antivirals**	0.6 (0.48-0.77)	<0.001
Atazanavir containing products	2.6 (0.84-8.1)	0.2-0.1
Efavirenz	0.1 (0.04-0.3)	<0.001
Dolutegravir	0 (N/A)	<0.001
Tenofovir containing products	0.8 (0.60-1.10)	0.975-0.2
Zidovudine containing products	1.1 (0.51-2.33)	0.995-0.975
Other antivirals	0.7 (0.35-1.27)	0.975-0.2
**Antibacterials**	1.4 (1.06-1.73)	0.02-0.01
Co-trimoxazole	3.7 (1.91-7.26)	<0.001
Gentamicin	0.2 (0.09-0.46)	<0.001
Ceftriaxone	2.7 (0.96-7.41)	0.1-0.05
Flucloxacillin	1.9 (0.75-4.22)	0.975-0.2
Amoxicillin containing products	1.2 (0.81-1.91)	0.975-0.2
Other antibacterial agents	1.6 (0.97-2.51)	0.1-0.05
**Analgesics**	8.5 (5.59-13.02)	<0.001
Paracetamol containing products	19.4 (9.90-38.18)	<0.001
Aspirin containing products	4.2 (0.47-37.66)	0.975-0.2
Ibuprofen	0.8 (0.31-1.91)	0.975-0.2
Tramadol	∞ (N/A)	0.05-0.025
Diclofenac	∞ (N/A)	<0.001
Other analgesics	0 (N/A)	0.2-0.1
**Antihypertensives**	1.3 (0.95-1.88)	0.2-0.1
Amlodipine containing products	2.0 (0.77-5.34)	0.975-0.2
Losartan containing products	1.4 (0.70-2.62)	0.975-0.2
Nifedipine	1.4 (0.5-3.95)	0.975-0.2
Furosemide	0.9 (0.41-2.15)	0.975-0.2
Methyldopa	0.6 (0.19-1.79)	0.975-0.02
Other antihypertensives	1.7 (0.9-3.34)	0.2-0.1
**Medical** **devices**	6.2 (3.72 -10.46)	<0.001
**Antiprotozoals**	1.4 (0.93-2.10)	0.2-0.1
Metronidazole	5.9 (4.93-7.12)	0.1-0.05
Artemisinin antimalarials	1.1 (0.52-2.12)	0.995-0.975
Sulfadoxine + Pyrimethamine	0.9 (0.06-14.9)	0.975-0.2
Aminosidine (Paromomycin)	∞ (N/A)	0.975-0.2
Secnidazole	0 (N/A)	0.975-0.2
Tinidazole	∞ (N/A)	0.975-0.2
**Gynaecological** **agents**	2.2 (1.39-3.52)	<0.001
**Antimycobacterial** **agents**	0.6 (0.36-0.86)	0.02-0.01
**Blood** **and** **perfusion** **solutions**	0.5 (0.29-0.72)	<0.001
**Anaesthetics**	10.1 (4.02-25.32)	<0.001
**Mineral** **supplements** **+** **vitamins**	0.7 (0.44-1.21)	0.975-0.2
**Drugs** **used** **in** **diabetes**	0.4 (0.17-0.88)	0.05-0.025
**Vaccines**	2.5 (0.96-6.33)	0.1-0.05
**Other** **therapeutic** **products**	2.1 (1.63-2.66)	<0.001

Note: “∞” represents “Infinity”, which occurs when the observed count for other medicines or MQRCs in the contingency Table 2 of the PvERS database is zero, leading to a disproportionately high outcome value

Table 6 highlights the reporting rates of sPQMs attributed to sTF (category 2) in the PvERS database. The data show that sTF were primarily reported from antineoplastic agents (20.2%; ROR:5.6; *p*-value:<0.001) and blood and perfusion solutions (2.1%; ROR:2.6; *p*-value:<0.001).

**Table 6:** Reporting rates of MQRCs in Kenya’s PvERS database attributed to sTF

**Therapeutic Class**	**ROR (95% CI)**	***p*-value (non-exact)**
**Antineoplastics**	5.6 (4.68-6.73)	<0.001
Imatinib	5.9 (4.93-7.12)	<0.001
Fluorouracil	0 (N/A)	0.975-0.2
Cyclophosphamide	1.2 (0.11-12.89)	0.975-0.2
Ifosfamide + Mesna	∞ (N/A)	0.2-0.1
Other antineoplastic agents	1.3 (0.39-4.57)	0.975-0.2
**Antivirals**	0.5 (0.38-0.64)	<0.001
Dolutegravir	5.0 (1.84-13.29)	<0.001
Tenofovir containing products	0.3 (0.19-0.41)	<0.001
Atazanavir containing products	0.2 (0.04-0.82)	0.05-0.025
Zidovudine containing products	0.5 (0.19-1.08)	0.2-0.1
Efavirenz	1.4 (0.72-2.59)	0.975-0.2
Other antivirals	0.5 (0.23-1.00)	0.1-0.05
**Antibacterials**	0.8 (0.64-1.05)	0.2-0.1
Gentamicin	4.1 (2.01-8.47)	<0.001
Co-trimoxazole	0.2 (0.07-0.41)	<0.001
Ceftriaxone	0.5 (0.18-1.37)	0.975-0.2
Flucloxacillin	0.7 (0.31-1.73)	0.975-0.2
Amoxicillin containing products	0.1 (0.65-1.53)	0.975-0.2
Other antibacterial agents	0.7 (0.43-1.14)	0.2-0.1
**Analgesics**	0.2 (0.10-0.23)	<0.001
Paracetamol containing products	0.1 (0.03-0.13)	<0.001
Diclofenac	0 (N/A)	<0.001
Tramadol	0 (N/A)	0.1-0.05
Aspirin containing products	0 (N/A)	0.2-0.1
Ibuprofen	1.7 (0.69-4.30)	0.975-0.2
Other analgesics	∞ (N/A)	0.1-0.05
**Antihypertensives**	0.6 (0.38-0.79)	0.002-0.001
Methyldopa	2.2 (0.71-6.69)	0.975-0.2
Furosemide	1.4 (0.59-3.16)	0.975-0.2
Nifedipine	0.3 (0.1-1.21)	0.2-0.1
Amlodipine containing products	0.3 (0.07-0.88)	0.05-0.025
Losartan containing products	0.4 (0.17-0.84)	0.025-0.02
Other antihypertensives	0.4 (0.19-0.83)	0.02-0.01
**Antiprotozoals**	0.9 (0.59-1.35)	0.975 -0.2
Metronidazole	0.8 (0.48-1.35)	0.975 -0.2
Artemisinin antimalarials	1.1 (0.54-2.20)	0.975 -0.2
Sulfadoxine + Pyrimethamine	1.4 (0.09-22.38)	0.975 -0.2
Aminosidine (Paromomycin)	0 (N/A)	0.975 -0.2
Secnidazole	∞ (N/A)	0.975 -0.2
Tinidazole	0 (N/A)	0.975 -0.2
**Blood** **and** **perfusion** **solutions**	2.9 (1.81-4.49)	<0.001
**Antimycobacterial** **agents**	1.8 (1.16-2.70)	0.02-0.01
**Gynaecological** **agents**	0.5 (0.33-0.85)	0.01-0.005
**Medical** **devices**	0.2 (0.10-0.29)	<0.001
**Anaesthetics**	0.1 (0.05-0.32)	<0.001
**Mineral** **supplements** **+** **vitamins**	1.4 (0.83-2.26)	0.975-0.2
**Drugs** **used** **in** **diabetes**	2.8 (1.27-6.34)	0.02-0.01
**Vaccines**	0.5 (0.20-1.34)	0.975 - 0.2
**Other** **therapeutic** **products**	0.5 (0.35-0.59)	<0.001
Note: “∞” represents “Infinity”, which occurs when the observed count for other medicines or MQRCs in the contingency Table 2 of the PvERS database is zero, leading to a disproportionately high outcome value		

Table 7 shows the sPQMs attributed to sADRs (category 3) in the PvERS database. The data show sADR were predominantly notified from antivirals (3.7%; ROR: 14.6; *p*-value < 0.001). Notably, tenofovir, efavirenz, zidovudine, dolutegravir, and other antivirals also exhibited high reporting rates, with RORs of 12.1, 16.7, 7.3, 8.4, and 13.6, respectively (all *p*-values < 0.001).

**Table 7:** Reporting rates of MQRCs in Kenya’s PvERS database attributed to adverse drug reactions

**Therapeutic class**	**ROR (95% CI)**	***p*-value (non-exact)**
**Antineoplastics**	0.1 (0.03-0.17)	< 0.001
Imatinib	0.1 (0.02-0.15)	<0.001
Fluorouracil	0 (N/A)	0.995-0.975
Cyclophosphamide	0 (N/A)	0.975-0.2
Ifosfamide + Mesna	0 (N/A)	0.975-0.2
Other antineoplastic agents	1.1 (0.14-8.35)	0.975-0.2
**Antivirals**	14.6 (10.48-20.27)	< 0.001
Tenofovir containing products	12.1 (8.44-17.28)	<0.001
Efavirenz	16.7 (8.01-34.86)	<0.001
Zidovudine containing products	7.3 (2.68-19.75)	<0.001
Dolutegravir	8.4 (3.06-23.19)	<0.001
Atazanavir containing products	4.6 (1.02-20.5)	0.2-0.1
Other antivirals	13.6 (6.45-28.46)	<0.001
**Antibacterials**	0.6 (0.33-1.08)	0.2-0.1
Amoxicillin containing products	0.2 (0.02-1.18)	0.1-0.05
Co-trimoxazole	1.5 (0.58-3.75)	0.975-0.2
Gentamicin	1.2 (0.35-3.76)	0.975-0.2
Flucloxacillin	0 (N/A)	0.975-0.2
Ceftriaxone	0 (N/A)	0.975-0.2
Other antibacterial agents	0.6 (0.19-1.92)	0.975-0.2
**Analgesics**	0.1 (0.01-0.43)	< 0.001
Paracetamol containing products	0 (N/A)	<0.001
Ibuprofen	0 (N/A)	0.975-0.2
Diclofenac	0 (N/A)	0.975-0.2
Tramadol	0 (N/A)	0.975-0.2
Aspirin containing products	3.4 (0.38-30.57)	0.975-0.2
Other analgesics	0 (N/A)	0.975-0.2
**Antihypertensives**	2.2 (1.31-3.71)	0.005-0.002
Losartan containing products	3.7 (1.58-8.47)	0.005-0.002
Furosemide	0 (N/A)	0.975-0.2
Amlodipine containing products	2.9 (0.85-10.21)	0.2-0.1
Nifedipine	3.9 (1.1-14.05)	0.1-0.05
Methyldopa	0 (N/A)	0.975-0.2
Other antihypertensives	2.2 (0.87-5.80)	0.2-0.1
**Antiprotozoals**	0.1 (0.02-1.04)	0.05-0.025
Metronidazole	0 (N/A)	0.1-0.05
Artemisinin antimalarials	0.5 (0.06-3.41)	0.975-0.2
Sulfadoxine + Pyrimethamine	0 (N/A)	0.975-0.2
Aminosidine (Paromomycin)	0 (N/A)	0.1-0.05
Secnidazole	0 (N/A)	0.1-0.05
Tinidazole	0 (N/A)	0.1-0.05
Antimycobacterial	1.1 (0.47-2.56)	>0.995
**Gynaecological** **agents**	0.3 (0.08-1.40)	0.2-0.1
**Medical** **devices**	0.4 (0.11-1.12)	0.1-0.05
**Blood** **and** **perfusion** **solutions**	0 (N/A)	0.05-0.025
**Anaesthetics**	0 (N/A)	<0.001
**Mineral** **supplements** **+** **vitamins**	1.0 (0.36-2.80)	0.975-0.2
**Drugs** **used** **in** **diabetes**	0.6 (0.08-4.20)	0.975-0.2
**Vaccines**	0 (N/A)	0.975-0.2
**Other** **therapeutic** **products**	1.0 (0.64-1.63)	0.975-0.2

### Sources of MQRCs within the Kenya healthcare system

This study examined the sources of MQRCs within the Kenya healthcare system, which is categorized into six hierarchical levels starting with the lowest primary health facilities and culminating in the national referral health facilities level [13]. Table 8 demonstrates that majority of MQRCs originated from government health facilities (60.0%; *n*=1659) and licensed private for-profit health facilities (26.5%; *n*=733). The MQRCs notifications from government health facilities were associated with sMQDs (39.1%; ROR:3.9; *p*-value< 0.001) as presented in Table 9. Notifications from licensed private for-profit health facilities primarily reported sTF (19.9%, ROR:7.1; *p*-value < 0.001) while licensed not-for-profit health facilities were mainly attributed to sADRs (0.8%; ROR:2.9; *p*-value < 0.001).

**Table 8:** Sources of medicine quality-related complaints from the Kenya healthcare system

Complaint origin	sMQDs		sTF		sADRs		Grand Total	
**Government** **health** **facility**	*n*=1082	39.1%	*n*=449	16.2%	*n*=128	4.6%	*n*=1659	60.0%
Hospital	*n*=822	29.7%	*n*=296	10.7%	*n*=108	3.9%	*n*=1226	44.3%
Dispensary/ medical clinic/ health centre	*n*=255	9.2%	*n*=153	5.5%	*n*=20	0.7%	*n*=428	15.5%
Importer/ distributor/ wholesaler	*n*=5	0.2%	*n*=0	0.0%	*n*=0	0.0%	*n*=5	0.2%
**Formal** **(Licensed)** **private** **for-profit** **health** **facility**	*n*=172	6.2%	*n*=549	19.9%	*n*=12	0.4%	*n*=733	26.5%
Hospital	*n*=48	1.7%	*n*=522	18.9%	*n*=9	0.3%	*n*=579	20.9%
Pharmacy/Chemist	*n*=70	2.5%	*n*=5	0.2%	*n*=0	0.0%	*n*=75	2.7%
Dispensary/ medical clinic/ health centre	*n*=43	1.6%	*n*=19	0.7%	*n*=2	0.1%	*n*=64	2.3%
Importer/ distributor/ wholesaler	*n*=11	0.4%	*n*=3	0.1%	*n*=1	0.0%	*n*=15	0.5%
**Formal** **(Licensed)** **not** **for-profit** **health** **facility**	*n*=61	2.2%	*n*=66	2.4%	*n*=23	0.8%	*n*=150	5.4%
Hospital	*n*=34	1.2%	*n*=48	1.7%	*n*=15	0.5%	*n*=97	3.5%
Dispensary/ medical clinic/ health centre	*n*=27	1.0%	*n*=18	0.7%	*n*=8	0.3%	*n*=53	1.9%
**Informal unlicensed facility**	*n*=126	4.6%	*n*=87	3.1%	*n*=12	0.4%	*n*=225	8.1%
Individual reporters	*n*=14	0.5%	*n*=2	0.1%	*n*=1	0.0%	*n*=17	0.6%
Unknown sources	*n*=112	4.1%	*n*=85	3.1%	*n*=10	0.4%	*n*=207	7.5%
Research Institution	*n*=0	0.0%	*n*=0	0.0%	*n*=1	0.0%	*n*=1	0.0%
**Grand** **Total**	*n*=1441	52.1%	*n*=1151	41.6%	*n*=175	6.3%	*n*=2767	100.0%

**Table 9:** Poor-quality medicine complaints based on their sources within the Kenya healthcare system

Complaint category	Disproportionality analysis measure	Government health facility	Licensed private for-profit health facility	Licensed not for-profit health facility	Informal unlicensed health facilities
**sMQDs**	ROR	3.9 (3.33-4.60)	0.2 (0.15-0.22)	0.6 (0.44-0.86)	1.2 (0.90-1.56)
	*p*-value	<0.001	<0.001	0.01-0.005	0.975-0.2
	No of reports	39.1%, *n*=1082	*n*=172, 6.2%	2.2%, *n*=61	4.6%, *n*=126
**sTF**	ROR	0.2 (0.18-0.25)	7.1 (5.86-8.60)	1.1 (0.80-1.55)	1.9 (1.43-2.48)
	*p*-value	<0.001	<0.001	0.975-0.2	<0.001
	No of reports	*n*=449, 16.2%	19.9%, *n*=549	2.4%, *n*=66	3.1%, *n*=87
**sADR**	ROR	1.9 (1.34-2.66)	0.2 (0.11-0.35)	2.9 (1.83-4.71)	0.8 (0.45-1.50)
	*p*-value	<0.001	<0.001	<0.001	0.975-0.2
	No of reports	*n*=128, 4.6%	0.4%, *n*=12	0.8%, *n*=23	0.4%, *n*=12

**Fig. 2:**
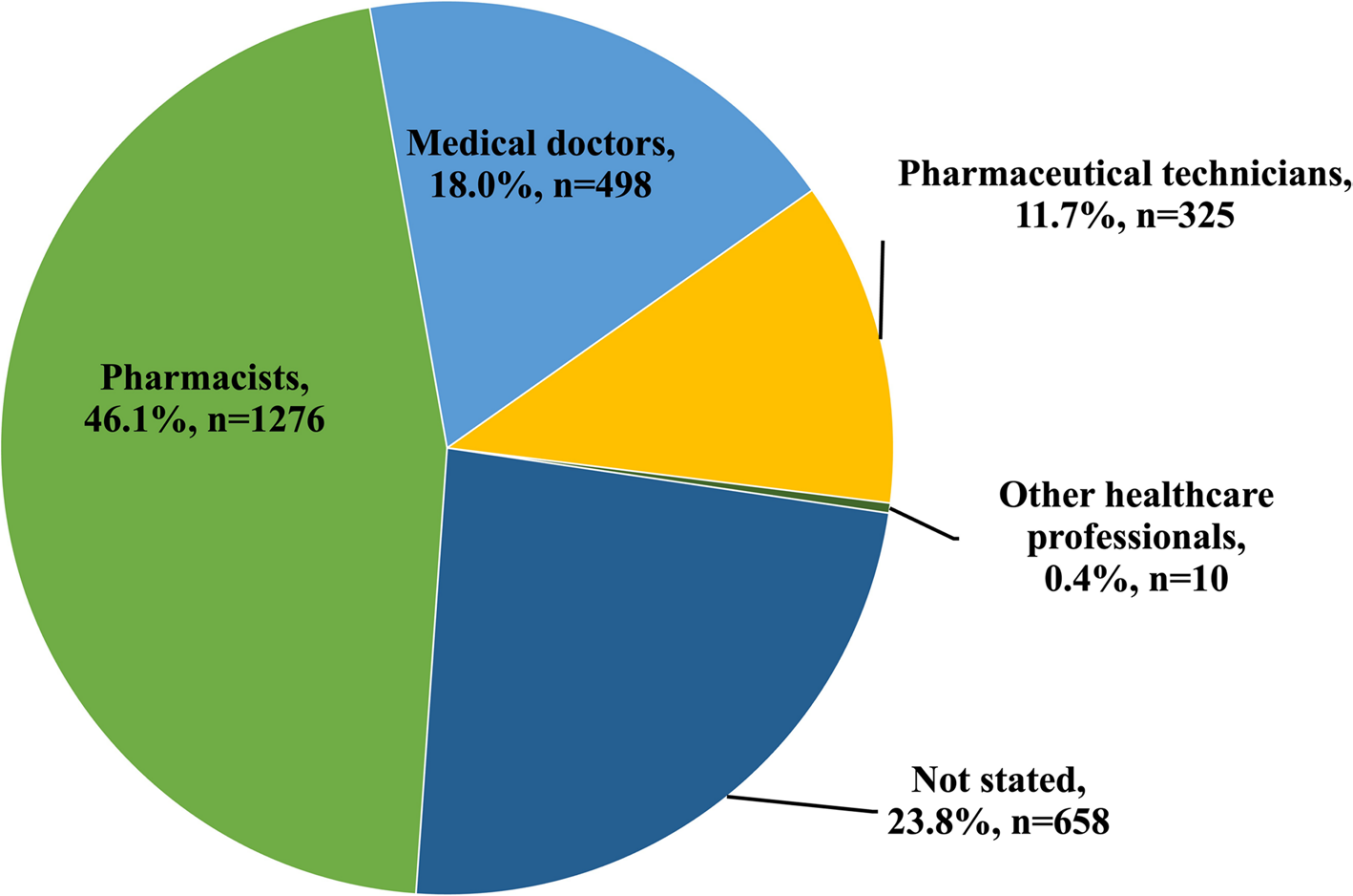
Representation of professional categories notifying complaints in Kenya

This study investigated the Kenya healthcare professionals responsible for reporting MQRCs due to their pivotal role in the medicine supply chain [13]. Among healthcare professional categories responsible for complaint notifications, pharmacists were predominant, followed by medical doctors, and pharmaceutical technicians, as depicted in Fig. 2. sMQDs were notified by pharmacists (34.4%; ROR:6.0; *p*-value:<0.001) and pharmaceutical technicians (9.9%; ROR:5.9; *p*-value:<0.001) and attributed to direct interaction with patients. On the other hand, medical doctors (13.5%; ROR:5.8; *p*-value:<0.001) reported mostly suspected therapeutic failures. A small proportion of complaints originated from other healthcare professionals, including clinical officers, nurses, and health information officers.

Evidently in Table 10, a substantial number of MQRCs (23.8%, *n*=658), mostly notified as suspected therapeutic failure (ROR:2.9; *p*-value:< 0.001) had not specified the professional category of the reporter, likely presumed to originate from anonymous members of the public and patients.

**Table 10:** Healthcare professionals categories reporting medicine quality-related complaints in Kenya’s PvERS database

Kenya healthcare professional	Disproportionality analysis measure	Poor medicine quality attribution		
		sMQDs	sTF	sADRs
**Pharmacists** 46.1%, *n*=1276	ROR	6.0 (5.10-7.102)	0.3 (0.23-0.32)	0 (N/A)
	*p*-value (non-exact)	< 0.001	< 0.001	< 0.001
	No. of reports	34.4%, *n*=952	11.7%, *n*=324	0
**Medical** **doctors** 18.0%, *n*=498	ROR	0.2 (0.19-0.30)	5.8 (4.64-7.22)	0 (N/A)
	*p*-value (non-exact)	< 0.001	< 0.001	< 0.001
	No. of reports	4.5%, *n*=124	13.5%, *n*=374	0
**Pharmaceutical** **technicians** 11.7%, *n*=325	ROR	5.9 (4.31-8.00)	0.2 (0.17-0.31)	0 (N/A)
	*p*-value (non-exact)	< 0.001	< 0.001	< 0.001
	No. of reports	9.9%, *n*=274	1.8%, *n*=51	0
**Other** **healthcare** **professionals** 0.4%, *n*=10	ROR	∞ (N/A)	0 (N/A)	0 (N/A)
	*p*-value (non-exact)	0.01-0.005	0.02 - 0.01	< 0.001
	No. of reports	0.4%, *n*=10	0	0
**Professional** **not** **stated** 23.8%, *n*=658	ROR	0.1 (0.06-0.10)	2.9 (2.38-3.41)	∞ (N/A)
	*p*-value (non-exact)	< 0.001	< 0.001	< 0.001
	No. of reports	2.9%, *n*=81	14.5%, *n*=402	6.3%, *n*=402

Note: “∞” represents “Infinity”, which occurs when the observed count for other medicines or MQRCs in the contingency Table 2 of the PvERS database is zero, leading to a disproportionately high outcome value

**Fig. 3:**
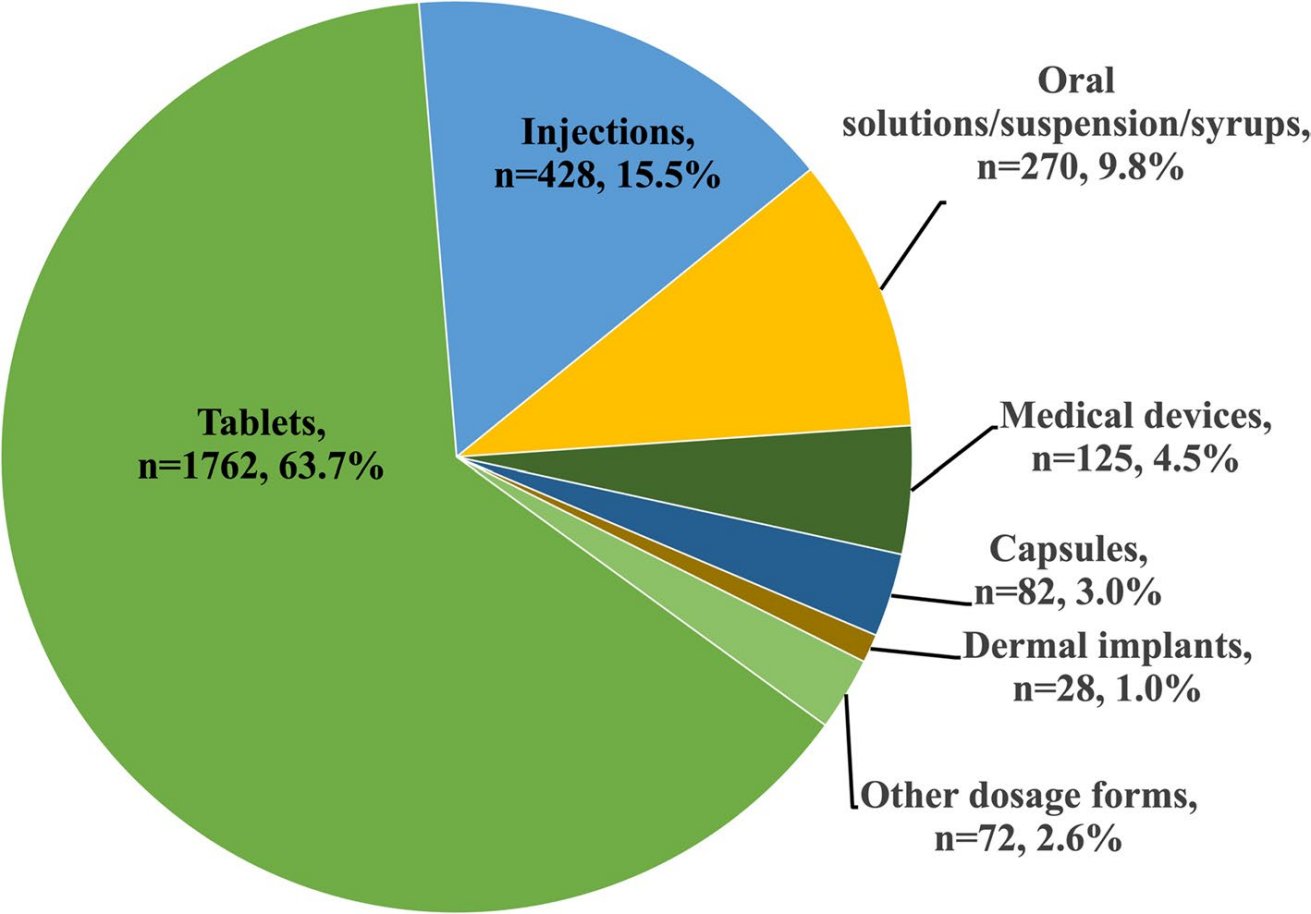
The types of dosage forms notified as MQRCs

This study investigated the dosage formulations notified as MQRCs. Figure 3 shows that medicines administered in oral dosage forms (76.9%, *n*=2127) were the most reported. Oral administered medications were the most reported in the database. The pharmaceutical dosage forms notified as MQRCs showed that tablet formulations (5.7%, ROR:6.1; *p*-value:< 0.001) were primarily notified as sADRs, as shown in Table 11. Conversely, sMQDs were reported from in oral solutions, suspensions, and syrups (7.0%, ROR:2.6; *p*-value:< 0.001) and medical devices (3.9%, ROR:6.2; *p*-value:< 0.001).

**Table 11:** Dosage forms notified as medicine quality-related complaints in the Kenya’s PvERS database

Dosage form	Disproportionality analysis measure	Poor-quality medicine attribution		
		sMQDs	sTF	sADRs
**Tablets** 63.7%, *n*= 1762	ROR	0.4 (0.34-0.48)	1.9 (1.57-2.17)	6.1 (3.64-10.31)
	*p*-value (non-exact)	< 0.001	< 0.001	< 0.001
	No. of reports	28.1%, *n*=777	28.9%, *n*=826	5.7%, *n*=159
**Injections** 15.5%, *n*= 428	ROR	1.4 (1.12-1.71)	0.9 (0.71-1.08)	0.3 (0.17-0.61)
	*p*-value (non-exact)	0.005 - 0.002	0.975 - 0.2	< 0.001
	No. of reports	9.1%, *n*=252	6.0%, *n*=166	0.4%, *n*=10
**Oral** **solutions/** **Suspensions/** **Syrups** 9.8%, *n*= 270	ROR	2.6 (1.94-3.37)	0.1 (0.02-0.09)	0 (N/A)
	*p*-value (non-exact)	< 0.001	< 0.001	< 0.001
	No. of reports	7.0%, *n*=194	2.7%, *n*=76	0
**Medical** **devices** 4.5%, *n*= 125	ROR	6.2 (3.72-10.46)	0.2 (0.10-0.30)	0.4 (0.11-1.12)
	*p*-value (non-exact)	< 0.001	< 0.001	0.1 - 0.05
	No. of reports	3.9%, *n*=108	0.5%, *n*=14	0.1%, *n*=3
**Capsules** 3.0%, *n*= 82	ROR	0.8 (0.51-1.23)	1.7 (1.06-2.56)	0 (N/A)
	*p*-value (non-exact)	0.975 - 0.2	0.05 - 0.025	0.05 - 0.025
	No. of reports	1.4%, *n*=38	1.6%, *n*=44	0
**Dermal** **implants** 1.0%, *n*= 28	ROR	1.2 (0.58-2.61)	0.9 (0.42-1.95)	0.6 (0.07-4.04)
	*p*-value (non-exact)	0.975 - 0.2	0.975 - 0.2	0.975 - 0.2
	No. of reports	0.6%, *n*=16	0.4%, *n*=11	0%, *n*=1
**Other** **dosage** **forms** 2.6%, *n*= 72	ROR	3.3 (1.89-5.80)	0.3 (0.18-0.60)	0.4 (0.10-1.71)
	*p*-value (non-exact)	< 0.001	< 0.001	0.975 - 0.2
	No. of reports	2.0%, *n*=56	0.5%, *n*=14	0.1%, *n*=2

### Impact of COVID-19 pandemic and geographical source of notified of MQRCs in Kenya

**Fig. 4:**
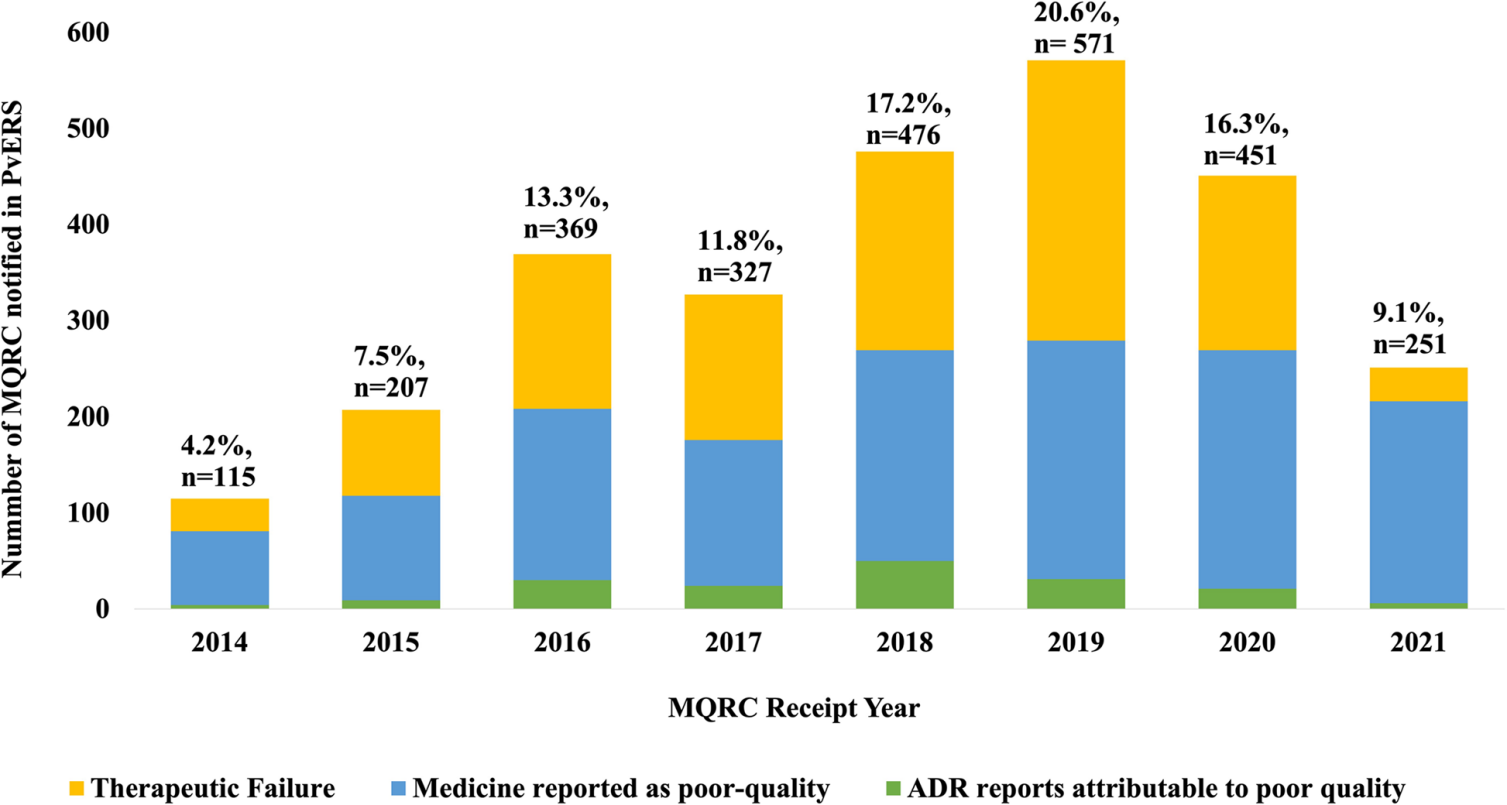
Impact of COVID-19 on notification of medicine quality-related complaints in Kenya’s PvERS database

This impact of the COVID-19 pandemic on the notification of MQRCs to the PPB exhibited a disruption in the notification of medicine quality complaints during this public health emergency. Figure 4 shows a decline in the number of MQRCs recorded in the PVERS database following the onset of the COVID-19 pandemic in March 2020.

The distribution of notified MQRCs in the PvERS database from 2014 to 2021 across various Kenyan counties are presented in Table 12. Nairobi exhibited the highest frequency of MQRCs notifications, followed by Kiambu, and Mombasa. Certain counties, including Garissa, Lamu, Trans-Nzoia, and Wajir, did not report any MQRCs during this period. Notably, counties in remote semi-arid locations, such as Turkana, Nyandarua, Isiolo, Kwale, Kitui, Elgeyo-Marakwet, Marsabit, Samburu, Tana River, and Mandera, reported fewer MQRCs notified in the PvERS database, reflecting lower healthcare infrastructure and possibly reduced access to reporting systems. A significant number of complaints lacked critical information necessary for the follow-up of MQRCs, such as the county name (9.1%, *n*=252).

**Table 12:** Medicine quality-related complaints notified in the PvERS database from 2014 – 2021 from Kenyan counties

Kenya county name	2014	2015	2016	2017	2018	2019	2020	2021	Total	Percentage
Nairobi	39	62	160	118	157	227	184	99	1046	37.8%
Kiambu	4	20	30	40	16	31	7	14	162	5.9%
Mombasa	8	11	8	20	13	22	17	7	106	3.8%
Kilifi	4	6	8	9	22	17	14	13	93	3.4%
Nakuru	6	11	10	4	11	14	18	10	84	3.0%
Kisumu	3	4	11	7	29	10	9	4	77	2.8%
Counties reports <2.6%	42	73	117	90	186	205	152	82	947	34.2%
Not Indicated	9	20	25	39	42	45	50	22	252	9.1%
Total	115	207	369	327	476	571	451	251	2767	100.0%

## Discussion

### Therapeutic classes and categories of notified as MQRCs

The findings in Fig. 1 showed a significant discrepancy in attention given to MQRCs, which is noticeably lower compared to sADEs within the context of the pharmacovigilance system in Kenya. This disparity can primarily be linked to the focus on sensitisation on monitoring the safety of medicines [21] rather than sPQMs. Table 3 show that branded medicines were associated with sMQDs possibly resulting from inherent characteristics and perceived reporting biases. Healthcare workers are likely to closely monitor specific aspects in branded medicines, thereby enhancing the detection and reporting of medicines with quality defects. For example, variations in the package appearance of a parallel-imported branded medicine may prompt notification due to perceived non-expected physical appearance, a difference that may be misjudged as suspected poor-quality. Conversely, generic medicines notified as sADRs arose from their high market prevalence, driven by affordability and expected established safety and efficacy profiles, increasing the likelihood of reporting.

The therapeutic classes notified in Table 4 align with Kenya’s Demographic and Health Survey of 2022 [22], reflecting a shift from primarily communicable diseases to non-communicable diseases as leading causes of morbidity and mortality in Kenya. The existence of poor-quality antibacterial [9] and antihypertensives [23] in Kenya has been documented and thus support the study findings. The rising prevalence of NCDs in Kenya can be imputed to sedentary lifestyle behaviours, increased tobacco and alcohol use, and poor nutrition [24]. Conversely, the reduction in communicable diseases is due to improvements in sanitation, diagnosis, access to effective treatments, and preventive vaccines [25]. While this shift does not explicitly pertain to sPQMs, it indirectly underscores the risk of compromised treatment effectiveness posed to individuals with NCDs and communicable diseases.

The reporting rates of sMQDs (category 1) from analgesics in Table 5 contradict the findings from the Kenya Drug Analysis and Research Unit pentad reports which showed a decline in the failure rate of tested analgesics [26]. The increased reporting of analgesics, specifically paracetamol-containing products, may be influenced by their overuse as an over-the-counter medication for pain management. Suspected poor-quality medical devices coupled with higher demand during the COVID-19 pandemic may have resulted due to lapses in manufacturing practices. The pandemic exacerbated shortages, hoarding, supply chain disruptions, and internet purchases of medical devices [27]. As a result, several poor-quality medical devices, such as rapid diagnostic test kits, gloves, and face masks, were reported in Kenya by the WHO [28] during this period. In general, the regulation of medical devices faces challenges, including neglect and lack of clear definition and scope [29]. Similar challenges exist in Europe despite the implementation of new regulations [30]. Harmonized definition and scope of medical devices among healthcare workers and regulatory agencies will enhance effective regulation. The increased reporting of suspected poor-quality gynaecological agents and anaesthetics may be associated with poor manufacturing and insufficient quality assurance systems.

In general, sMQDs manifest as alterations in physical appearance, chemical composition, or packaging of a product, requiring proactive identification. These pose a challenge for healthcare workers, who often lack the technical capacity to identify them during clinical practice. Notably, the existing literature [31] primarily focuses on troubleshooting on sMQDs during the actual medicine manufacturing process *in situ*, rather than facilitating on-the-field passive monitoring. Additionally, literature on medicine quality monitoring and authentication [32], typically rely on poorly designed medicine sampling surveys and testing for estimation of prevalence [10]. This study also revealed that some notified MQRCs had incomplete data and errors, emphasizing the need for standardized data capture terminology to enhance collection. To the best of our knowledge, no comprehensive list of standard terminology describing potential sMQDs commonly observed during clinical practice for oral dosage forms exists. This study proposes the development of a patient-friendly standard terminology to be incorporated into the Medical Dictionary for Regulatory Activities (MedDRA) [33] as shown in Table 13. This standardized terminology aims to facilitate passive notification of sMQDs by patients and healthcare workers in the field. Consistent data collection using this terminology would enable integration with machine learning and artificial intelligence technologies, resulting in faster and more accurate signal detection of sMQDs.

**Table 13:** Description, causes, and consequences of common medicine with quality defects in substandard and falsified oral dosage forms

Observed medicine with quality defects	Prevalent dosage forms	Defect description	Probable cause	Consequence
Caking (agglomeration)	Suspension and Powder for oral solution / suspension	Coherent solid lumps or masses formed	Environmental degradation	Decreased drug efficacy, non-uniform, and non-homogenous dosing to patients
Capping and lamination	Tablets	Partial or whole tablet separation into two or more layers	Poor manufacturing practice	Inelegant appearance, patient unacceptability, and possible inaccurate dosing
Chipping or breaking	Tablets	Edge breakage/ splitting or fissure during handling and transportation	Poor manufacturing practice	Incorrect drug administered and patient unacceptability
Cracking	Tablets	Fine small breakages on the tablet surface of tablets	Poor manufacturing practice	Inelegant appearance, patient unacceptability, and possible inaccurate dosing
Incomplete package	Tablets, capsules	Empty blister bubbles with missing dose unit	Poor manufacturing practice	Patient unacceptability
Leakage	Liquid dosage forms	Outflow of internal contents	Poor manufacturing practice	Drug loss and incorrect drug administered
Mislabelling	All dosage forms	Incorrect details or advice	Poor manufacturing practice	Medication errors
Mottling (Colour change)	Tablets, Capsules and Powder for oral solution / suspension	Non-uniform or unequal distribution or variation in the shade or colour	Environmental degradation and poor manufacturing practice	Patient unacceptability
Moulding	Tablets, Capsules and Powder for oral solution / suspension	Microbial contamination and spoilage	Environmental degradation and poor manufacturing practice	May infect a patient, drug degrading, may cause toxicity and patient unacceptability
Odour change	All dosage forms	Unpleasant smell or unusual gas accumulation	Environmental degradation	Drug degrading may cause toxicity and patient unacceptability.
Phase inversion (cracking)	Emulsions	Separation into constituent phases	Environmental degradation	Inelegant appearance, patient unacceptability, and possible inaccurate dosing
Powdering	Tablets	Particles erode on mechanical shaking or during handling	Poor manufacturing practice	Incorrect drug administered and patient unacceptability
Sticking, picking, or binding	Tablets, Capsules and Powder for oral solution / suspension	Tablet glued to the package surface.	Poor manufacturing practice	Patient unacceptability
Taste change	All dosage forms	Unusual and unpleasant savour	Environmental degradation	Patient non-adherence to therapy
Uneven Splitting	Tablets	Irregular breaking at the scoring	Poor manufacturing practice	Non-uniform dosing to patients
Unusual stains pots	Tablets, Capsules and Powder for oral solution / suspension	Unusual observable light or dark spots or smudges on the surface	Poor manufacturing practice	Patient unacceptability

The significant reporting rates of sTF in Table 6 for antineoplastic agents, particularly imatinib, highlight the vulnerability of this therapeutic class. In 2017, the WHO issued a rapid alert on falsified antineoplastic agents in East Africa [34] supporting these findings. The link of antineoplastic agents to sTF potentially stems from complexity and deviations of formulation and manufacturing processing, late stage of disease diagnosis and resistance [35, 36] patient non-adherence [37], and individual patient factors [38] requiring a further comprehensive understanding. Among other frequently notified therapeutic classes associated with sTF, antibacterial agents accounted for 4.0% (*n*=111), with gentamicin showing a significant association (1.1%; ROR:4.1; *p*-value:<0.001), likely attributed to drug resistance. Similarly, no demonstrable association was found for antivirals and sTF, except for dolutegravir (0.7%; ROR:5.0; *p*-value:<0.001) which may be from drug resistance [36], bio-inequivalence and inadequate dosing. The presence of sTF undermine patient adherence to pharmacotherapy, foster mistrust in prescribed medications, impede disease management, and exacerbate health outcomes thereby diminishing confidence in the healthcare system. Typically, other factors such as misdiagnosis, inappropriate medicine selection or dosage, medication errors, drug-drug interactions [39], and subjective brand preferences influenced by unethical medical promotional practices [13] may result to clinical-related sTF that are not necessarily associated with MQRCs. According to literature [40], the currently used causality assessment tools utilised for determining the causes of sTF, prioritize excluding clinical causes without investigating medicine quality thus delaying prevention, detection, and response to suspected SF medicines, potentially impacting patient outcomes adversely. This study accentuates the imperative to modify the Vaca González et al. (2013) [40] causality assessment algorithm by expanding the number of questions and reorganizing their sequence to give precedence to review the medicine quality-related factors before scrutinizing intrinsic and extrinsic patient-related clinical factors. Implementation of such a modified approach in clinical settings will significantly contribute to enhanced medication safety and improved patient care.

The high reporting rates of sADRs for antivirals in Table 7 may be due to increased awareness of well-documented side effects among patients undergoing chronic antiretroviral therapy for HIV/AIDS treatment. This is complemented with enhanced pharmacovigilance sensitization of healthcare workers in public health programs by the PPB. Furthermore, pharmacogenetic drug-drug interactions, particularly with concomitant use of antiretroviral therapy with medications such as rifampicin and isoniazid [41, 42], may contribute to this association, altering drug concentrations, resulting in treatment failure and drug resistance.

### Sources of MQRCs within the Kenya healthcare system

The PvERS database, despite being freely available, exhibits sPQMs reporting gaps between the public and private health sectors as shown in Tables 8 and 9. The differences in reporting between the Kenya public and private health sectors are multifaceted. Challenges include lack of feedback, mistrust, limited capacity and incentives for reporting. Moreover, public healthcare personnel receive more extensive pharmacovigilance training compared to their private sector counterparts, who often are required to self-finance their training. The underreporting from primary health facilities is likely due to a lack of awareness and an underestimation of the impact of sPQMs within Kenya's healthcare system. Current pharmacovigilance sensitization initiatives primarily target training higher health facility levels healthcare workers [43], overlooking those in lower primary health facilities and non-healthcare professionals. This approach assumes that higher health facilities are the primary interactors with medicines in the supply system, a notion which is untrue. While focusing on healthcare workers in higher health facility levels is essential, information often fails to reach lower primary health facilities and non-healthcare professionals, hindering vigilance and response to sPQMs. Comprehensive sensitization across the healthcare system is crucial to enhance surveillance and response to sPQMs. Existing educational materials lack explanations about sPQMs, their risks, and the importance of notification tailored to non-pharmaceutical personnel. Developing targeted training materials is essential to increase reporting rates, especially from the public and healthcare workers in primary health facilities. Conspicuously, the PvERS database lacked notifications from informal unlicensed health facilities, despite studies indicating presence of SF medicines in this sector [44]. Patients using unlicensed health facilities often self-medicate and rely on out-of-pocket expenditures for more affordable inferior quality medicines. Reporting barriers are likely due to unawareness, unfriendly reporting systems, and culturally sensitive apprehensions such as privacy preservation and the stigma associated with reporting to regulatory authorities. These findings are consistent with the study carried out by Güner and Ekmekci (2019) who found that familiarity with the pharmacovigilance reporting system increases reporting [45]. Future research should focus on identifying barriers to reporting and establishing a formal system to handle reports from unlicensed health facilities to promote a culture of transparency and accountability.

The source of complaints within Kenya’s healthcare system affirmed the role, and expertise of pharmacists in identifying and reporting issues, especially sMQDs as shown in Fig. 2. The association of medical doctors with reporting of sTFs in Table 10 may be due to their frontline role in observing patient-related issues crucial for the safety and effectiveness of medical interventions.

The dosage formulations preferred by patients as shown in Fig. 3 were orally administered medications which aligns with existing literature [46] and attributed to their convenient administration, ease of use, non-invasiveness, accurate dosing, and high patient compliance and adherence [47–49]. However, they are prone to be substandard and falsified due to their ease of transportation and online availability [6]. Tablet formulations are often reported as sADRs in Table 11 possibly stemming from imprecise dosing, weight inconsistency, and the presence of toxic by-products from degradation. These findings highlight the need for focused oversight to orally administered medications formulations.

### Impact of COVID-19 pandemic and geographical sources of notified MQRCs in Kenya

These results in Fig. 4 on the impact of COVID-19 pandemic to MQRCs notification were surprising given that reports indicate that the exacerbated the public health problem of poor-quality medicines during the same period [50, 51]. These results imply that some Kenya patients may have unknowingly consumed poor-quality medications and blamed the effects to COVID-19. Multiple factors may have contributed to the underreporting of MQRCs during the COVID-19 pandemic, including panic purchasing of medications, the spread of distorted information about benefits of certain medicines, and a shift in healthcare-seeking behaviour by increased usage of medicines purchased through the internet. Additionally, changes in medication usage patterns, heightened anxiety levels, family priorities, a lack of resilient business continuity strategies, and limited resources may have all played a role in exacerbating underreporting. It is essential to emphasize that during public health emergencies, the primary focus tends to be on collecting health disease surveillance information rather than monitoring the quality of medicines. The lack of an active surveillance system designed to continuously monitor the utilization of medicines during such public health crises is of public health concern. This study proposes utilization of specific tracer oral medications identified through a predefined checklist of commonly expected poor-quality issues as proposed in Table 13 to facilitate improved detection of sPQMs during public health emergencies. Such a system may incorporate rapid non-destructive analytical testing technologies to identify suspicious medicines quickly, followed by confirmatory laboratory-based testing [52]. This approach offers a crucial solution in times of a public health crisis, thereby safeguarding public health and enhancing overall medication safety.

Table 12 highlights the challenges in MQRCs reporting across Kenyan counties. Urban areas like Nairobi, Kiambu and Mombasa, characterized by higher health professional density and heightened awareness of reporting systems showed higher higher MQRCs notifications. Conversely, remote and semi-arid counties reported fewer MQRCs, suggesting potential barriers related to low awareness, high workload, limited resource availability (including the internet or personnel), and logistical challenges. Reporting is often perceived to be a less urgent activity, particularly in regions characterized by geographic seclusion. Addressing these disparities will require targeted efforts to enhance awareness, streamline reporting procedures, and provide adequate resources in underserved regions, thereby improving the overall nationwide vigilance of MQRCs.

### Study limitations

The study focused solely on auditing subjective and qualitative MQRCs notified as ICSR within Kenya’s PvERS database, which cannot be directly attribute to poor-quality medicines such as results from chemical analyses in a quality control laboratory. If results from laboratory analyses had been included, they would have been considered as representative of the country’s prevalence of poor-quality medicines. Additionally, while the study aimed to analyse crucial information from the PvERS database, it did not conduct a data quality audit of the available data, reports, and documents pertaining to suspected poor-quality medicines reported in Kenya.

## Conclusion

The study quantitatively characterized MQRCs to evaluate their effectiveness for passive identification of suspected poor-quality medicines in the Kenya market. The approach complements the traditional survey methods by facilitating risk-based regulatory prioritization of suspected poor-quality medicines for targeted sampling and testing in Kenya and similar LMICs. The findings revealed an existing disparity in Kenya’s pharmacovigilance system whose focus is ADEs rather than sPQMs. Branded medicines are closely monitored for quality defects, while generic medicines, though more prevalent, show higher reporting rates for sADEs. This emphasizes the need for a more balanced approach integrating both sPQMs alongside sADEs in the pharmacovigilance system.

Therapeutic classes of notified MQRCs supported studies showing a shift from communicable to non-communicable diseases, reflecting changing morbidity and mortality in Kenya. Oral medicines, particularly tablets for both communicable and non-communicable diseases were identified as high-risk dosage forms, warranting heightened regulatory monitoring. High reporting rates of sTF for antineoplastic agents highlight their vulnerability due to formulation complexities. Tools for assessing causality in sTF cases should prioritize medicine quality over clinical factors for quicker identification of sPQMs. The COVID-19 pandemic exacerbated reporting of poor-quality medical devices, exposing regulatory challenges due to neglect and lack of clear scope and definitions.

The study highlighed the crucial role healthcare workers, particularly pharmacists, in identifying and reporting sPQMs despite a critical gap identified in the global literature. The study proposes the inclusion of patient-friendly standardized terminology in MedDRA to improve passive notification and standardize data capture of sPQMs by healthcare workers in the field.

Significant underreporting from primary health facilities, remotely areas, and informal unlicensed health facilities due to various reporting barriers, necessitating tailored sensitization initiatives to encourage reporting of MQRCs. Additionally, the study demonstrated the challenges in monitoring medicine quality during public health emergencies, stemming from focus on disease surveillance rather than medicine quality monitoring. An active surveillance system using commonly reported medicines and a predefined checklist of commonly reported MQRCs for rapid analytical testing is proposed to bolster medicine control during public health crises.

Overall, this study provides robust evidence and valuable insights to inform regulatory practices and strengthen pharmacovigilance and post-market surveillance across all levels of Kenya’s healthcare system, with direct applicability to other LMICs.

## Data Availability

The data that support the findings of this study are available from the Kenya’s national regulatory authority’s the Pharmacy and Poisons Board. The processed, analysed, discussed, and presented data in the publication belong to De Montfort University. The data presented in this study are available on request from the corresponding author.

## Abbreviations

sADEs: Suspected Adverse drug events
sADRs: Suspected Adverse drug reactions
APIs: Active pharmaceutical ingredients
COVID-19: Coronavirus disease
HIV/AIDS: Human Immunodeficiency Virus/ Acquired immunodeficiency syndrome
ICSRs: Individual case safety reports
LMICs: Low- and middle-income countries
MedDRA: Medical Dictionary for Regulatory Activities
sMQDs: Suspected Medicine with quality defects
MQRCs: Medicine quality-related complaints
PPB: The Pharmacy and Poisons Board
sPQMs: Suspected Poor-quality medicines
PvERS: The Pharmacovigilance Electronic Reporting System
ROR: Reporting Odds Ratio
SF: Substandard and falsified
sTF: Suspected Therapeutic failure
WHO: The World Health Organization
